# Himalayan Nettle *Girardinia diversifolia* as a Candidate Ingredient for Pharmaceutical and Nutraceutical Applications—Phytochemical Analysis and In Vitro Bioassays

**DOI:** 10.3390/molecules25071563

**Published:** 2020-03-29

**Authors:** Shyam Sharan Shrestha, Stefania Sut, Irene Ferrarese, Serena Barbon Di Marco, Gokhan Zengin, Michele De Franco, Deepak Raj Pant, Mohamad Fawzi Mahomoodally, Nicola Ferri, Noemi Biancorosso, Filippo Maggi, Stefano Dall Acqua, Sangeeta Rajbhandary

**Affiliations:** 1 Central Department of Botany, Tribhuvan University, 44613 Kirtipur, Kathmandu, Nepal; shyamsharan999@gmail.com (S.S.S.); drpant.agbot@gmail.com (D.R.P.); s.rajbhandary@cdbtu.edu.np (S.R.); 2 DAFNAE, Department of Agronomy, Food, Natural Resources, Animals and Environment, Agripolis Campus, University of Padova, 35020 Legnaro (PD), Italy; stefania_sut@hotmail.it; 3 Department of Pharmaceutical and Pharmacological Sciences, University of Padova, Via Marzolo 5, 35131 Padova, Italy; irene.ferrarese@unipd.it (I.F.); serena.barbondm@gmail.com (S.B.D.M.); miky_defra@hotmail.com (M.D.F.); nicola.ferri@unipd.it (N.F.); noemi.biancorosso@unipd.it (N.B.); 4 Department of Biology, Science Faculty, Selcuk University, Campus, 42130 Konya, Turkey; gokhanzengin@selcuk.edu.tr; 5 Institute of Research and Development, Duy Tan University, Da Nang 550000, Vietnam; mohamadfawzimahomoodally@duytan.edu.vn or; 6 Department of Health Sciences, Faculty of Science, University of Mauritius, Réduit 80837, Mauritius; 7 School of Pharmacy, University of Camerino, Via Sant’Agostino 1, 62032 Camerino, Italy; filippo.maggi@unicam.it

**Keywords:** phytosterols, LC-MS, polyphenols, NMR, cytotoxicity, LDLR, low-density lipoprotein receptor

## Abstract

*Girardinia diversifolia*, also known as Himalayan nettle, is a perennial herb used in Nepal to make fiber as well as in traditional medicine for the treatment of several diseases. To date, phytochemical studies and biological assays on this plant are scarce. Thus, in the present work, the *G. diversifolia* extracts have been evaluated for their potential pharmaceutical, cosmetic and nutraceutical uses. For this purpose, detailed phytochemical analyses were performed, evidencing the presence of phytosterols, fatty acids, carotenoids, polyphenols and saponins. The most abundant secondary metabolites were β- and γ-sitosterol (11 and 9% dw, respectively), and trans syringin (0.5 mg/g) was the most abundant phenolic. Fatty acids with an abundant portion of unsaturated derivatives (linoleic and linolenic acid at 22.0 and 9.7 mg/g respectively), vitamin C (2.9 mg/g) and vitamin B2 (0.12 mg/g) were also present. The antioxidant activity was moderate while a significant ability to inhibit acetylcholinesterase (AChE), butyrilcholinesterase (BuChE), tyrosinase, α-amylase and α-glucosidase was observed. A cytotoxic effect was observed on human ovarian, pancreatic and hepatic cancer cell lines. The effect in hepatocarcinoma cells was associated to a downregulation of the low-density lipoprotein receptor (LDLR), a pivotal regulator of cellular cholesterol homeostasis. These data show the potential usefulness of this species for possible applications in pharmaceuticals, nutraceuticals and cosmetics.

## 1. Introduction

*Girardinia diversifolia* (Link) Friis (Urticaceae) is a stout, erect, perennial herb, 25–200 cm tall, with a perennial rootstock. The aerial parts are armed with numerous slender stinging hairs and the leaves have 3–5 deep lobes, and are saw-toothed with bristles. The flowers are yellowish, clustered in a panicle; the male ones are white and borne in lower axillary panicles; the female ones are grouped in upper bristly axillary and terminal panicles. Flowers appear from July to September and fruits from September to November [1]. The plant is found on a clump with many stems, whose bark contains strong, smooth and light fibers, largely used for textiles in Nepal. *G. diversifolia* occurs abundantly in different parts of Nepal, notably in hill forests on moist and damp soil, at altitudes of 1200–3000 m; it is also found in northern India, Bhutan, Sri Lanka, eastward of central China, Myanmar, Malaysia, Indonesia and Africa. In Nepali, the plant is known as “allo” or “chalnesisno” as its leaf is broad and palm-like. In English it is commonly known as “Himalayan nettle” due to the presence of stinging hairs that cause irritation on the skin when touched. 

*G. diversifolia* is largely used in traditional medicine for the treatment of several diseases such as gastric disorders, chest pain [2], rheumatism, tuberculosis [3], headache, joint aches [4], diabetes [1,5], asthma, gastritis, headache, joint pain, tuberculosis [6], gonorrhea and delivery problems [5]. Other traditional uses are related to the treatment of bone fracture, internal injury and blood purification [7]. It is also famous for the uses of the bast fiber of its bark, for making varieties of clothing, ropes, mats, sacks and other domestic implements. In this context, it is one of the most important non-timber forest products used for income generation among rural communities in the Himalaya region of Nepal for their livelihood. Young leaves and inflorescences are cooked as a green vegetable. Roasted seeds are consumed pickled [4]. Other in formation about this specie are available in supplementary informations. 

There is a renewed interest in the study and evaluation of botanicals as active ingredients of herbal medicines and pharmaceutical, cosmetic and nutraceutical preparations. In particular, the area of nutraceuticals, naturally derived mixtures that are claimed to improve health and to prevent diseases [8], can be an innovative area of research for the discovery of useful remedies. In this regard, Nepalese medicinal and aromatic plants can be a valuable source for the discovery of bioactive extracts and constituents.

A literature search revealed limited information about the phytochemical content of *G. diversifolia*. Sterol derivatives were isolated from plants collected in Kenia, namely β-sitosterol, 7-hydroxysitosterol and 3-hydroxystigmast-5-en-7-one, and the antimicrobial activity was determined [9]. 

Thus, with the aim to consider *G. diversifolia* as a starting material for the preparation of new nutraceutical or pharmaceutical products, in this paper, we evaluated this plant as a source of bioactive phytochemicals. The work was performed combining LC-DAD-MS^n^ (liquid chromatography–diode array detector–tandem mass spectrometry) and GC-MS (gas chromatography–mass spectrometry) analysis with in vitro bioassays focused on some key enzymes involved in global health problems. Some of them are related to the central nervous system, namely acetylcholinesterase and butyrilcholinesterase. Others (e.g., tyrosinase) are involved in skin disorders highlighting potential use in cosmetics. Furthermore, due to possible importance of phytochemicals acting on metabolic syndrome, key enzymes in managing glucose levels, such as α-amylase and α-glucosidase, were taken into account. To complete the pattern of considered bioactivities, metal chelating and antioxidant capacity of the extract were performed, and preliminary cytotoxic activity was assayed on three different tumor cell lines. Finally, the capability to interfere with the synthesis of LDL receptors was studied in hepatocarcinoma cells. All the data were corroborated by detailed phytochemical fingerprinting, offering an overview of chemical composition and preliminary bioactivity of this species.

## 2. Results

### 2.1. Phytochemical Analysis

**Figure 1:**
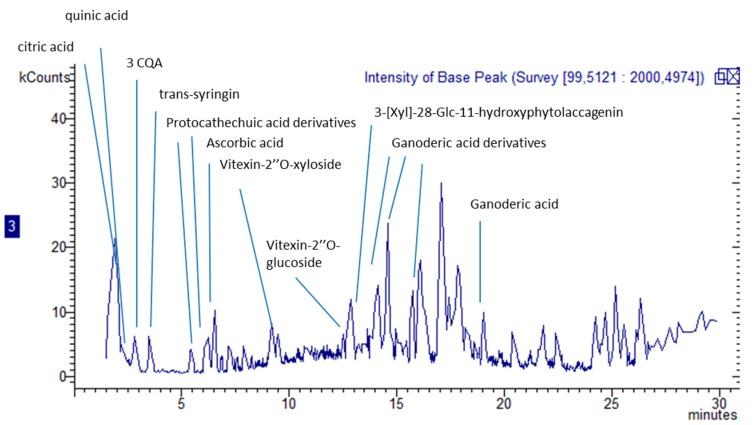
LC-MS chromatogram in negative ion mode; some of the identified peaks reported in Table 1 are highlighted.

**Figure 2:**
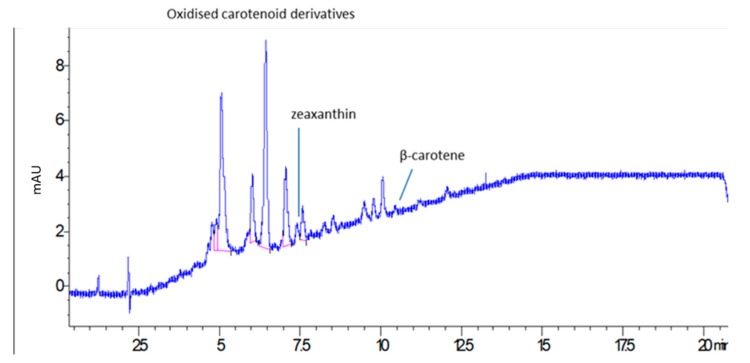
LC-DAD chromatogram related to the analysis of carotenoids and derivatives (425 nm).

For screening purposes, the extraction of secondary metabolites was performed on dried plant material, using methanol as the solvent due to its ability to extract both lipophilic and hydrophilic constituents. Due to the different chemical constituents that can be present in plant material, different analytical methods were used. *G. diversifolia* extract was analyzed by LC-APCI-MS^n^ (liquid chromatography–atmospheric pressure chemical ionization–tandem mass spectrometry)and LC-DAD-ESI-MS^n^ (liquid chromatography–diode array detector–electrospray-tandem mass spectrometry) to obtain information on the phytochemical composition of the plant material sampled in Nepal. A specific method, using C-18 stationary phase coupling, with chromatography equipped with atmospheric pressure chemical ionization (APCI) mass spectrometry (MS), was used for the identification and quantification of terpenoids and phytosterols. A C-30 stationary phase coupled with a diode array detector (DAD) and APCI-MS was used to assess the presence and to quantify carotenoid derivatives. Furthermore, a C-18 column and electrospray (ESI-MS) were used to identify more hydrophilic constituents, such as, for example, phenolics and saponins. The identified compounds are reported in Table 1 and Table 2, along with the amounts of the most abundant compounds. Figure 1 and Figure 2 represent the obtained chromatograms.

Phytosterols and triterpenes, with β-sitosterol, γ-sitosterol, campesterol, fucosterol and sitostanol as the most abundant constituents, were detected in the plant extract. To confirm the structure of γ-sitosterol, this compound was isolated, and the structure was elucidated by comparing the obtained 1D and 2D NMR data with the literature [10,11].

**Figure 3:**
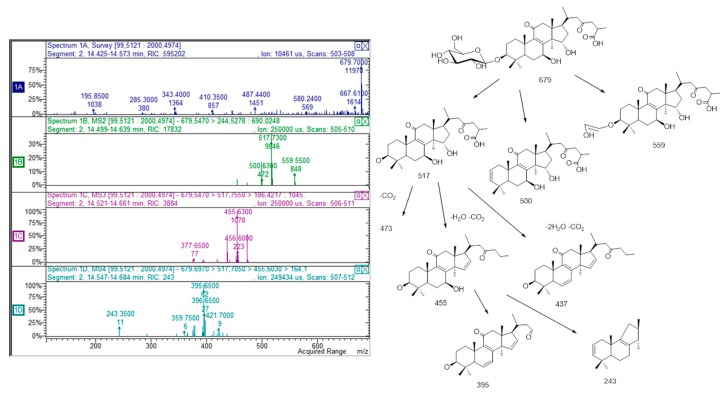
Proposed fragmentation scheme and MS^n^ spectrum of derivative *m*/*z* 679, tentatively assigned to a glycosidic derivative of ganoderic acid.

Overall, the phytosterols content is notable, considering their amount corresponded to 26% of total methanol extract. Analysis was also performed to assess the presence of carotenoids. The results revealed a limited amount of such compounds in the prepared extract, with a total content of 0.47 mg/g. In comparison with the reference standard, a low amount of β-carotene and zeaxanthin were detected, while other derivatives that were assigned to oxidized products of carotenoids, mostly β-carotene epoxide, were observed and tentatively identified on the basis of their MS spectra and comparison with the literature [12]. The LC-DAD at 450 nm is reported in Figure 3. The presence of degradation products of carotenoids may be due to extraction procedures or drying of plant material.

Polar constituents were analyzed by LC-DAD-ESI-MS^n^, and some organic acids, namely citric and quinic acids were detected; the most abundant was ascorbic acid, assessed at 2.9 mg/g. These compounds are quite common in vegetal sources. A detectable but not quantifiable amount of gluconic acid was also observed.

Some peaks ascribable to flavonoids were detected as iso-orientin, vitexin and its derivatives, and kaempferol glucoside [13,14]. Other minor phenolic derivatives, mostly caffeoylquinic acid esters [15], syringic, protocatecuic and sinapoyl acid derivatives [13] were also present. In positive ion mode, traces of complex malvidin glycoside linked to catechin units was observed [16].

LC-MS^n^ data made it possible to reveal several peaks that present *m/z* values, and a fragmentation pattern that can be ascribed to saponins: in particular, a peak at retention time of 13 min with *m*/*z* 841 and fragmentations suggesting that the structure can be assigned to 3-[Xyl]-28-Glc-11-hydroxyphytolaccagenin [17]. An isomer of this compound presenting a different pattern of fragmentation is observed at retention time of 11.8 min and was tentatively assigned to an iridoid derivative [18]. Furthermore, different peaks presenting *m*/*z* values and fragmentation patterns that suggest the presence of ganoderic acid derivatives were detected. In particular, from the peak at retention time of 18.9 min with molecular ion [M – H]^−^ at *m*/*z* 517 we can observe MS^2^ fragments due to the elimination of water (−18 amu) and subsequent loss of water and CO_2_(−62 amu). Similar behavior is also observed for three other peaks at retention times of 14.3, 14.9 and 16.3 min, presenting *m*/*z* values of 679. The MS^n^ spectrum, as well as a proposed scheme of fragmentation, is reported in Figure 3 as an example. These data, when compared with the literature [14,19], suggest the presence of ganoderic acid derivative in *G. diversifolia* samples although at very low concentration. These metabolites are typical of *Ganoderma* fungi but have recently been reported in *Rubus* fruits, leaves and flowers [14]. To the best of our knowledge, no other papers have reported the presence of such derivatives in the Urticaceae family. We can suggest that symbiotic fungi can be grown in association with plants and that these metabolites can be derived from the fungal metabolism. However, further investigation should be performed to assess the origin of these metabolites in the Himalayan nettle. Finally, a low but detectable amount of vitamin B2 was measured.

**Figure 4:**
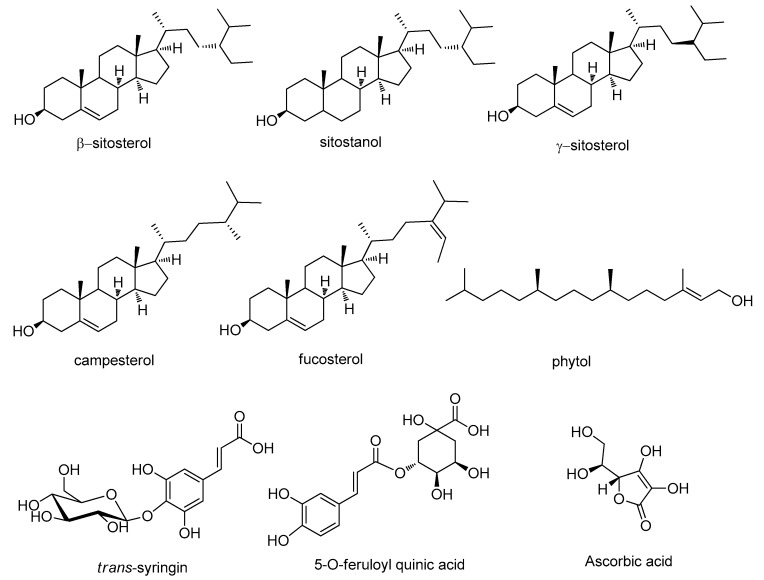
Structure of the most abundant constituents found in the *G.diversifolia* extract.

Due to the large amount of lipophilic compounds, GC-MS analysis was also performed after derivatization with methanol to study fatty acid composition and to reveal the presence of other lipophilic constituents. The results are reported in Table 3. The methanolic extract of *G. diversifolia* contained a 13% lipidic fraction, including 46% saturated derivatives and 32% unsaturated fatty acids. The most abundant fatty acid was palimitic acid, while linoleic acid was the most abundant one (22 mg/g) among unsaturated fatty acids. Phytol was the main constituent of the lipophilic fraction (27 mg/g). Results are summarized in Table 3. Figure 4 presents the structures of the most abundant constituents of the *G. diversifolia* extract.

### 2.2. In Vitro Bioassays

The results indicate significant AchE inhibitory activity for *G. diversifolia* extract as reported in Table 4. The phytochemical analysis indicates the presence of some non-alkaloidal inhibitors. The search for non-nitrogen-containing AchE and BuChE inhibitors is of interest, since alkaloids are known for their activity in this regard but their use leads to unpleasant side effects [20,21]. Furthermore, non-alkaloidal inhibitors probably have different types of interaction with the target enzyme due to the lack of a charged part, thus offering the opportunity to find other pharmacological properties [20].

AchE predominates in the healthy brain, with BuChE considered to play a minor role in regulating brain acetylcholine levels [22]. Both enzymes represent legitimate therapeutic targets for ameliorating the cholinergic deficit considered to be responsible for the neurological decline occurring in Alzheimer’s disease (AD). Tyrosinase is a main enzyme in the synthesis of melanin and its inhibition was found to be significant, showing potential usefulness of the extract for cosmetic uses and skin related diseases.

A-Amylase and α-glucosidase are enzymes related to carbohydrate digestion and their inhibition could help to control postprandial glucose levels in diabetes mellitus patients. Considering the α-amylase and α-glucosidase inhibition, the observed activities can be considered moderate, with values around 1 mmol acarbose equivalent (ACAE)/g for α-amylase inhibition and 5.4 mmol ACAE/g for α-glucosidase, demonstrating limited activity of *G. diversifolia* extract as reported in Table 4.

The tests on the antioxidant and total phenolic constituents are in agreement with the obtained data from the chromatographic measurements and are summarized in Table 5. The levels of polyphenols appear to be moderate and also the antioxidant effects. The extract exhibited scavenging abilities on DPPH (2,2-diphenyl-1-picrylhydrazyl) (14.37 mg TE/g extract) and ABTS (2,2’-azino-bis(3-ethylbenzothiazoline-6-sulfonic acid ) (28.33 mg TE/g extract). Reducing power reflects the electron-donation ability of the extract in CUPRAC (cupric reducing antioxidant capacity) (105.27 mg TE/g extract) and FRAP (ferric ion reducing antioxidant power) (42.63 mg TE/g extract) assays. In addition, the metal chelating activity (25.70 mg EDTAE/g) appears to be significant, showing potential usefulness in the protection of metal-based reactions producing free radicals.

Thus, taking into account the phytochemical composition and the in-vitro-enzyme-inhibitory properties and antioxidant capacity of *G. diversifolia* methanolic extract, we observed a significant effect on AchE and BuChE. Due to the low amount of polyphenols, we can suggest that this activity may be related to the high content of phytosterols. In a previous paper, stigmasterol was considered as an insecticidal constituent of the extract of the plant Chromolaena odorata or Eupatorium odoratum. Authors also reported that the phytosterol mixture was a better AchE inhibitor than its pure compound on C. quinquefasciatus AchE [23]. Other authors reported non-activity of stigmasterol in a mice model and in an vitro test at a dose of 0.3–30 µM (0.123 mg/L, 12.3 mg/L) [24], while other authors reported AchE inhibition with IC_50_ of 644 µM (265 mg/L) [25].

Additionally, β-sitosterol has been evaluated for its ability to inhibit AchE, and IC_50_ of 24.1 ± 0.7 µg/mL (58.1 ± 1.6 µM) was reported as showing significant ability to inhibit this enzyme [26]. Furthermore, other authors have reported that β-sitosterol was an efficient inhibitor of BuChE, with an IC_50_ value of 0.56 µM and also presenting significant selectivity compared to AchE (IC_50_14.57 µM). Authors explained this activity by studying the docking positions of side chains and hydroxyl groups at the enzyme’s active site [26].

Another compound that is present in a high quantity in *G. diversifolia* extract is phytol and its presence can also be related to the observed inhibitory properties, both on AchE and BuChE. A previous paper reported, for phytol that was isolated from marine organisms, an IC_50_ of 2.7 and 5.8 µM, respectively. The significant activity was corroborated by molecular docking studies that suggest that phytol is capable of forming strong interactions with Arg177, which is a residue outside the binding pocket of AchE [27]. Thus, the contribution of phytol to the *G. diversifolia* extract inhibitory activity on the two cholinesterase should be taken into account due to the high levels observed (2.7%) in the extract.

Considering the more polar constituents, syringin was reported to be active, having IC_50_ values of 31.47 ± 2.2 and 52.77 ± 0.12 µM, respectively, for AchE and BuChE [28], and showing significant selectivity on the latter enzyme. Thus, also, trans-syringin that is present in the methanol extract at 0.5mg/g can be considered to explain the efficacy of *G. diversifolia* inhibitor properties on these enzymes.

The presence of phytosterols, phytol and syringin make the extract of *G. diversifolia* a promising ingredient for nutraceuticals targeted for neuroprotection. Considering the lipophilic portion, a recent paper demonstrated an efficient neuroprotective role of phytosterols against high-cholesterol diet-induced cognitive deficits in aged rats [29].

Related to the tyrosinase activity, in this case the presence of high levels of phytosterols can also be related to the observed effect. A previous paper showed a significant effect for γ-oryzanol in cellular melanogenesis owing to its inhibition of tyrosinase enzyme activity and reduction of MITF and target genes in the PKA-dependent pathway [30].

### 2.3. Cytotoxic Activity

To obtain further information on the potential bioactivity of *G. diversifolia*, we considered in vitro cellular models. As a starting point, preliminary cytotoxicity tests of *G. diversifolia* methanolic extract were performed on three tumor cell lines, 2008 (ovarian cancer), BxPC3 (pancreatic cancer) and Huh7 (hepatocarcinoma). The *G. diversifolia* extract was tested on three different tumor cell lines, showing a significant cytotoxic effect on BxPC3 (IC_50_ = 12.5 µg/mL) and Huh7 cells (IC_50_ of 7.2 ± 2.5 µg/mL).

Furthermore, due to the complex phytochemical composition of the extract, we performed further fractionation, and cyclohexane, dichloromethane and ethyl acetate soluble fractions of methanolic extract were obtained in order to separate lipid constituents from the more hydrophilic compounds. 

**Figure 5:**
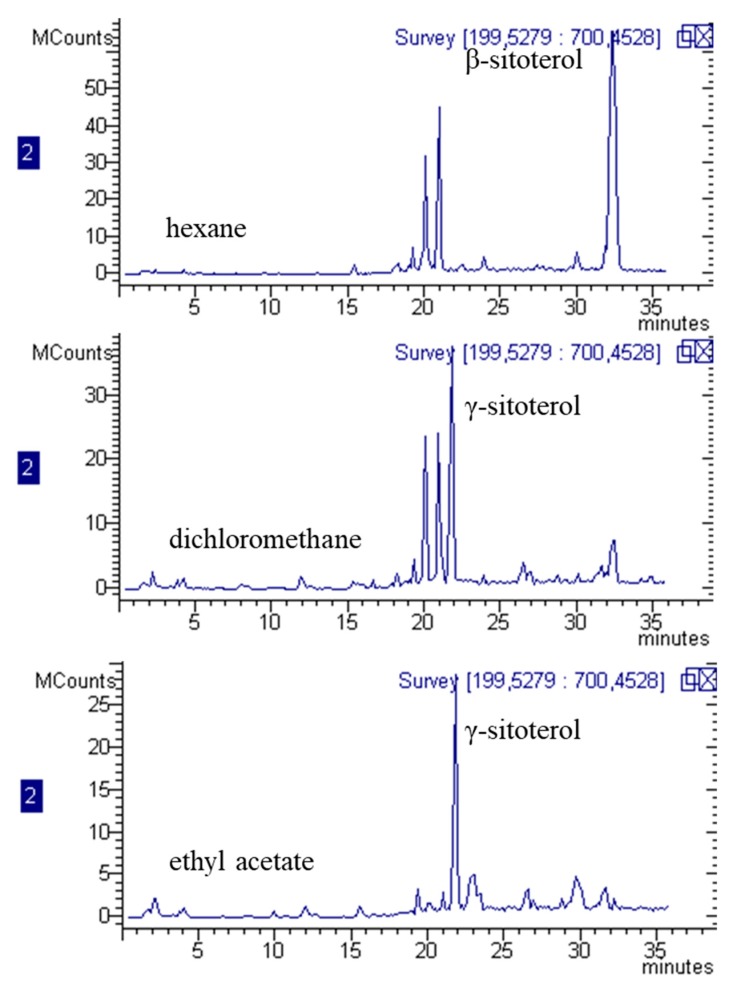
HPLC-MS chromatograms (base peak ion) of the three fractions obtained from the methanol extract by liquid–liquid partition, hexane, dichloromethane and ethyl acetate.

Exemplificative LC-MS chromatograms related to the phytosterol content are reported in Figure 5.

The lipophilic portion showed differences in phytosterol composition, with β-sitosterol being more concentrated in the hexane fraction and γ-sitosterol in the other ones; phenolic constituents were not detectable in these three extracts. Quantitative data for the most abundant lipophilic secondary metabolites derivatives is reported in Table 6.

**Figure 6:**
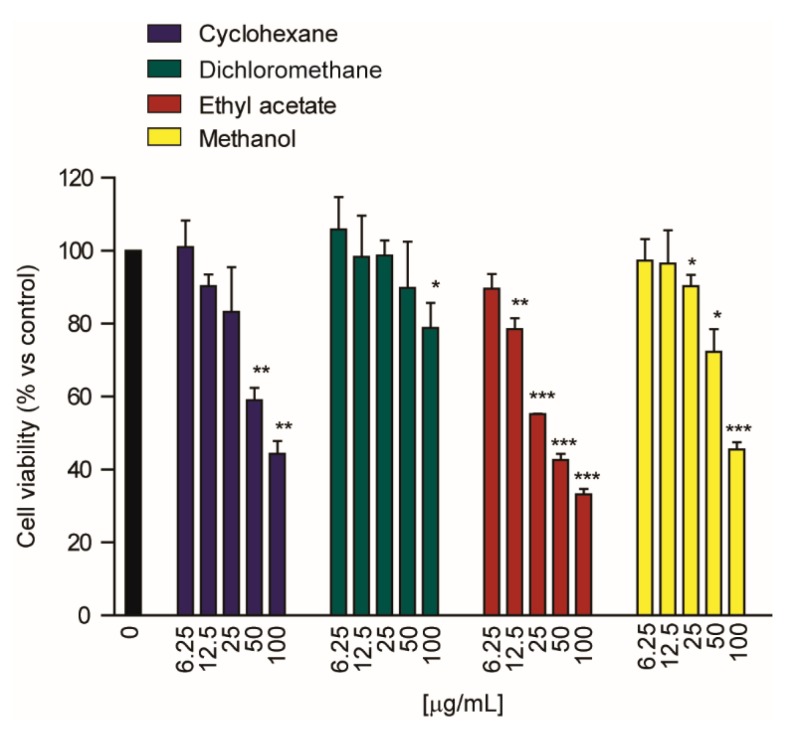
Cytotoxic effect of different solvent extracts of *G. diversifolia* on the Huh7 cell line. * *p* < 0.05; ** *p* < 0.01; *** *p* < 0.001. Student’s t-test.

The cytotoxic activity of the three fractions was determined using the Huh7 cells due to the lower observed IC**_50_.** The methanolic extract showed the most potent cytotoxic effect in Huh7 cells with an IC_50_ of 7.2 ± 2.5 μg/mL, compared to 19.9 ± 1.5 and 38.8 ± 1.2 μg/mL for ethyl acetate and cyclohexane extracts, respectively (Table 7 and Figure 6). Finally, dichloromethane extracts showed a significant effect of cell viability in the Huh7 cell line only at concentrations of 0.1 mg/mL (Figure 6); however, the IC_50_ value could not be determined, since the extent of the effect was less than 50%. Thus, these preliminary data showed that for the Huh7 cells the whole phytocomplex is more cytotoxic than the fraction enriched in lipophilic constituents.

### 2.4. Effect on LDLR Expression in Hepatoma Cell Line Huh7

**Figure 7:**
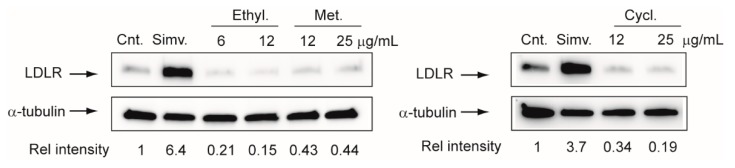
Effect of different solvent extracts of *G. diversifolia* on LDLR expression in Huh7 cell line.

To assess one possible mode of action in hepatocarcinoma cells, a test considering the ability of *G. diversifolia* extracts to induce the synthesis of LDLR in hepatocyte was performed. As shown in Figure 7, all different extracts of *G. diversifolia* showed a significant reduction of LDLR expression after 72 h of exposure. Considering the strong limitations of this in vitro analysis, it is conceivable to predict that *G. diversifolia* extract would not have significant lipid-lowering properties but may profoundly alter the intracellular cholesterol homeostasis. Tumor cells internalize cholesterol from circulating LDL through the LDLR on the cell surface, and it has been hypothesized that disruption of LDLR leads to intertumoral cholesterol imbalance and improvement of chemotherapy efficiency [31]. In addition, LDL-R expression is significantly increased in hepatocarcinoma, whereas it is less expressed in the adjacent liver tissue [32]. This evidence indicates that the extracts of *G. diversifolia* may exert their cytotoxic effects by reducing the expression of LDLR. In addition, in hepatocarcinoma, the increased levels of LDLR have been associated with the downregulation of Proprotein Convertase Subtilisin/Kexin Type 9 (PCSK9), a new pivotal pharmacological target for controlling hypercholesterolemia [33]. Further studies will be required in order to address this hypothesis.

Human hepatoma cells Huh7 were incubated for 72 h with indicated concentrations of *G. diversifolia* extracts, and LDLR reductase protein expression was evaluated by western blot analysis. α-Tubulin was used as a loading control. Densitometric readings were evaluated using the ImageLab^TM^ software and expressed as relative intensity (Rel. Intensity) compared with control. Cnt.: Control; Simv. Simvastatin (5 µM); Ethyl.: Ethyl acetate; Met.: methanol; Cycl. Cyclohexane. 

Fractionation of the extract with lipophilic solvent made it possible to obtain three fractions, but the total methanolic extract was the most active on Huh7 cells. The observed cytotoxic effect could be explained by the presence of phytosterols, such as β-sitosterol, γ-sitosterol, fucosterol and flavonoids. Previously published research showed that β-sitosterol exhibited significant ability on different cancer cell lines [34]. Furthermore, cytotoxic and proapoptotic properties of β-sitosterol were observed against leukaeima cells [35]. As previously reported, fucosterol extracted from Sargassum angustifolium marine algae showed cytotoxicity on colon carcinoma, colorectal adenocarcinoma and breast ductal carcinoma cell lines [36]. However, phenolic compounds also contribute to the toxicity. Recently, vitexin-2-O-xyloside’s antiproliferative properties have been studied, and this compound presented toxicity against CaCo2 cells and its activity was increased by coadministration of betalains [36]. However, considering the complex chemical nature of Himalayan nettle extract, further experiments are needed to purify and test specific compounds and fractions in order to deeply investigate the potential cytotoxic effect and the possible mode of action.

## 3. Discussion

*G. diversifolia* methanolic extract contains a high amount of phytol and phytosterols while phenolics, carotenoids, saponins are in a limited amount. Furthermore, the plant contains vitamin C and vitamin B2. The structures of the most abundant constituents are reported in Figure 4. The overall phytochemical composition suggests that the phytocomplex may be useful in the preparation of nutraceuticals. In particular, the results obtained in the chemical bioassays showed significant inhibition of cholinesterase. A previous paper indicated that phytosterols can have inhibitory properties on AChE and BuChE [24,29,37,38] and phytol has also been studied in this regard [24]. Previous published literature showed that phenolic constituents can act as inhibitors of these enzymes [39], thus suggesting that multiple compounds in the phytocomplex of *G. diversifolia* may be the responsible for the observed activity. Regarding the tyrosinase activity, literature data indicate that the phytosterol fraction indicated as oryzanol has significant inhibitory properties on this enzyme [30].

Regarding the antioxidant properties, the *G. diversifolia* methanolic extract exhibited moderate effects as revealed by different assays. Similar to enzyme inhibitory results, the observed antioxidant properties may be linked to non-phenolic antioxidants such as phytosterols, vitamin C, phytol and carotenoids. In some recent papers, important antioxidant mechanisms of non-phenolic compounds have been reported [38,40,41] and this is in accordance with our approach. Metal chelating ability reflects the inhibition of production of free radicals in the Fenton reaction and some non-phenolic compounds such as ascorbic acid, polysaccharides and peptides [42,43] have this ability. 

The *G. diversifolia* methanolic extracts were tested for cytotoxicity against three tumor cell lines, and the most potent cytotoxic effect was observed in Huh7 cells (IC_50_ of 7.2 ± 2.5 µg/mL). Extract fractionation reduced the cytotoxicity, thus indicating that the whole mixture presented a higher effect than the obtained fractions. Considering the phytochemical composition of the methanol extract, the cytotoxic activity can be ascribed to the overall phytocomplex, and in fact, a previous published paper indicates cytotoxic activity for phytosterols, fucosterol and flavonoids [34,35,36]. These different compounds may act by different modes of action. In this regard, our study considered a possible mode of action; in fact we observed a significant reduction of LDLR expression after 72 h of exposure and this effect can profoundly alter intracellular cholesterol homeostasis. Thus, the LDLR reduction can be considered as a possible mode of action to explain the observed cytotoxic effect on hepatocarcinoma cell lines. 

The overall results make it possible to have a deeper knowledge of the phytochemical composition of Himalayan nettle, and show its usefulness for cosmetic, pharmaceutical and nutraceutical applications.

## 4. Materials and Methods

### 4.1. Plant Material

Shoot tips of *Girardinia diversifolia* were collected from SindhuliGadi, Sindhuli, elevation range 1332–1394, GPS coordinates 27° 16’ 43” N and 85° 57’ 31” E, central part of Nepal. Plant materials were identified and authenticated with the KATH herbarium, Godawari, Lalitpur, Nepal.

The herbarium specimens were prepared following standard herbarium techniques (Bridsonand and Forman, 1992). The plants were dried carefully by proper pressing and the herbarium was prepared after mounting on the herbarium sheets. They were crosschecked with the herbarium, deposited at National Herbarium and Plant Laboratories, Godavari, Lalitpur (KATH), and submitted to TUCH (Tribhuvan University Central Herbarium), Kirtipur. Ethnobotanical information was collected from the literature. The working samples were collected and dried after making into small pieces. The sample was air dried over 2–3 weeks inside the room. The plant was grinded into fine powder with the help of an electric grinder. 

### 4.2. Extraction

The dried powder of plant material was subjected to extraction using methanol through percolation with intermittent sonication. An quantity of 10 g of plant powder was extracted three times with 100 mL of methanol so that the ratio of solvent in volume (mL) to the weight (g) of plant material would be 10:1. The solution was then subjected to intermittent sonication for 2 h, i.e., continuous cycle of sonication at 30 kHz for 30 min (with 10 min interruption). After the completion of the cycle, the solution was filtered with Whatman no.1 filter paper (Whatman Ltd., Kent, UK) and the filtrate was then subjected to evaporation at reduced pressure in a rotary evaporator (IKA RV 10, Milan, Italy). The concentrated extract thus obtained was transferred to clean, dried and weighed glass vials. The obtained extract was indicated as a methanol extract, and yield was 14%. The resulting dried extract was then sealed and stored at 4 °C until use. For the fractionation of the total methanol extract, the dried residue was dissolved in a mixture of 9:2 water: methanol and partitioned with cyclohexane, then dichloromethane then ethyl acetate. Organic solvents were collected and dried with sodium sulphate and then dried in rotavapor. Yields were 18%, 8%, 6% for cyclohexane, dichloromethane and ethyl acetate respectively.

### 4.3. HPLC-DAD-APCI-MS of Phytosterols, HPLC-DAD-APCI for Parotenoids, and HPLC-DAD-ESI-MS for Phenolic and Saponins

The extracts were analyzed using different methods. The instrumentation was an Agilent 1260 chromatograph (Santa Clara, CA, USA) equipped with a 1260 diode array detector (DAD), and a Varian MS-500 ion trap mass spectrometer equipped with ESI and APCI ion sources. At the end of the column, one “T” splitter separated the flow rate: half of the liquid was split to DAD and half to Agilent/Varian MS-500 ion trap mass spectrometer. UV-Vis spectra were acquired in the range of 190–400 nm. 

MS spectra were recorded using ESI in positive and negative ion mode, and APCI in positive mode. A turbo data-dependent scanning (TDDS) instrument function was used to acquire mass fragmentation pathways of the main ionic species. 

To specifically analyze phytosterols and terpene alcohols in *G. diversifolia*, ethyl acetate was used as a solvent and 100 mg of plant material was extracted using 30 mL of solvent. The liquid was filtered and the solvent was concentrated, and the volume was adjusted to 5 mL.

For the terpene and phytosterol constituents, an Agilent ZORBAX Eclipse XDB-C18 column (3.0 mm x 150 mm, 3.5 µm) was used as stationary phase. The mobile phase was water (0.1% formic acid) (A), acetonitrile (B) and methanol (C). A gradient program was used as follows: [0 → 8th min: A:B:C (2:95:3) → A:B:C (2:95:3) 8 → 10th min: A:B:C (2:95:3) → A:B:C (2:0:98) 10 → 28th min: A:B:C (2:0:98) → A:B:C (2:0:98) 28 → 30th min: A:B:C (2:0:98) → A:B:C (2:95:3) 30 → 35th min: A:B:C (2:95:3) → A:B:C (2:95:3)]. The flow rate was 0.4 mL/min, injection volume was 10 µL and the temperature was set at 30 °C. MS spectra were recorded in the range of *m*/*z* 100–2000, using an APCI ion source operating in positive ion mode. A turbo data-dependent scanning (TDDS) instrument function was used to acquire mass fragmentation pathways of the main ionic species. Identification of compounds was obtained based on comparison with the literature and reference compounds, when available. As standards, known solutions of β-sitosterol (176 µg/mL) and stigmasterol (185.6 µg/mL) were used.

Compound quantification was obtained with the method of calibration curve: β-sitosterol was used as the external standard for β-sitosterol and γ-sitosterol quantification, while stigmasterol was used for fucosterol. Calibration curves were as follows: y = 0.63x + 0.2705 (R^2^ = 0.99611) for β-sitosterol; y = 2.1153x − 15.216 (R^2^ = 0.9949) for stigmasterol.

For the analysis of polar constituents, such as polyphenol and saponins, Synergi Polar-RP (Phenomenex, Italy Bologna) was used as the stationary phase (3.0 × 150 mm; 4 micron); water, and 1% formic acid (A) and methanol (B), were used as eluents. The gradient began using 95% of solvent A and went to 25% of A in 15 min, then 0% A in 20 min. The flow rate was 0.4 mL/min. DAD chromatograms and UV-VIS spectra were acquired in the range of 200–650 nm. MS spectra were recorded in negative or in positive ion mode in the 50–2000 Da range, using an ESI ion source. Quantification of compound was obtained using DAD for hydroxycinnamic acid derivatives (chlorogenic, caffeic, syringic) chlorogenic acid solution was used (100–1 μg/mL) as a reference standard and the used wavelength was 330 nm. The calibration curve was Y = 1.325x + 0.236 (R^2^ = 0.9989). For flavonols, vitexin solutions (20–1 μg/mL) were used at 350 nm and the calibration curve was Y = 12.3x + 0.325. For saponins, escin was used as a reference compound and a 205 nm wavelength was used (100–10 μg/mL) Y = 0.325x + 0.023 (R^2^ = 0.9986).

For the analysis of carotenoids, a YMC carotenoid column (4.6 × 300 mm, 5 micron) was used as the stationary phase. Mobile phases were methylterbuthyl ether/methanol 90/10 (A) and methanol (B). The gradient started with 10% A and in 12 min reached 100% A. The flow rate was 1.3 mL/min. β-Carotene and zeaxanthin were used as a reference, preparing solution in the range 40–0.5 μg/mL. For the acquisition of MS spectra, APCI was used in positive ion mode, acquiring spectra in the *m/z* range of 365-1000 amu in TDDS ion mode.

### 4.4. GC-MS Analysis

For GC-MS analysis of the fatty acid content, a sample of *G. diversifolia* methanolic extract was derivatized with MeOH in presence of H_2_SO_4_, leading to the esterification of fatty acids to fatty acid methyl esters, which offer excellent stability for GC analysis. A quantity of 139.1 mg of the extract was added, with 15 mL of MeOH, 1 mL of CH_2_Cl_2_, 3 drops of H_2_SO_4_ and 25.7 mg of methylpentadecanoate (Sigma Aldrich, St. Louis, MO, USA), used as internal standard. The mixture was heated under reflux for 1 hour then cooled in an ice bath. A liquid–liquid partition was performed with 10 mL of water and 5 mL of diethyl ether, then the organic phase was collected and dried. The residue was re-dissolved with 1.5 mL of diethyl ether and put in vial. GC-MS analysis was performed through an Agilent 7820A coupled with a single quadrupole mass spectrometer Agilent 5977B MSD, using a HP88 (60 m × 0.25 mm, 0.2 µm film thickness) stationary phase. Helium was the carrier gas, with a column head pressure of 14.1 psi. The flow rate through the column was 1.19 mL/min. The injector was set at 300 °C with a split ratio of 20:1, the split flow was 23.9 mL/min and 1 µL injections were made. The temperature gradient started with a 120 °C initial temperature with a linear increase to 240 °C at 3 °C/min. The total run time was 55 min. MS spectra were recorded in the range of *m*/*z* 40–650, using an EI ion source operating in positive ion mode.

### 4.5. Isolation of Phytoconstituents 

Part of the methanolic extract (18 g) was separated through a silica gel column chromatography, using 1 L of a mixture of dichloromethane and methanol at 95:5, then 500 mL of 90:10, 500 mL at 80:20, 500 mL at 50:50, and finally 500 mL with 100% methanol as the mobile phase. Different fractions were obtained and analyzed by TLC (5% MeOH in CH_2_Cl_2_) and pooled on the basis of chromatographic behavior obtaining 85 fractions. 

Fractions 11–13(250 mg) and 14–16 (450 mg) presented significant spots, so they were pooled (750 mg) and separated through silica gel column chromatography, using 2 L of dichloromethane. Obtained fractions were analyzed through TLC, and the ones presenting similar behavior were collected and dried. Further purification was performed by preparative TLC (silica gel 60 F-254). In this way, two fractions were obtained and further purified by preparative TLC, allowing the isolation of β-sitosterol (32 mg) and γ-sitosterol (11 mg) respectively, which were identified on the basis of their NMR and MS measurements.

### 4.6. Bioassays

To obtain the total level of phenolic in the extract, colorimetric assay was used as described in our previous paper [44]. Gallic acid (GAE) was used as one standard, and the result was expressed as equivalent of gallic acid (mg GAE/g extract).

The antioxidant potential of the extracts was evaluated by phosphomolybdenum, antiradical (DPPH and ABTS), reducing power (FRAP and CUPRAC) and ferrous chelating assays as described [44]. Trolox equivalents were used for the expression of antioxidant activities. EDTA was employed as a reference compound for the metal chelating assay. The key enzymes inhibition activity of the extracts against AChE ((E.C. 3.1.1.7), from *Electrophorus electricus* (electric eel), Sigma-Aldrich, Darmstadt, Germany), BuChE ((E.C. 3.1.1.8), from equine serum, Sigma-Aldrich), tyrosinase ((E.C.1.14.18.1), from mushroom, Sigma-Aldrich), α-glucosidase ((E.C. 3.2.1.20), from *Saccharomyces cerevisiae*, Sigma-Aldrich) and α-amylase ((E.C. 3.2.1.1), from porcine pancreas, Sigma-Aldrich) were measured using the protocols previously reported [44]. Galanthamine (GALAE, for cholinesterases), kojic acid (KAE, for tyrosinase) and acarbose (ACAE, for amylase and glucosidase) were used as standard inhibitors in the enzyme assays.

### 4.7. Cell Cultures

A human pancreatic (BxPC3) carcinoma cell line was obtained by American Type Culture Collection (ATCC, RocKville, MD, USA). A total of 2008 human ovarian carcinoma cells were kindly provided by Prof. A. Marverti (Dept. of Biomedical Science of Modena University, Modena, Italy). Cell lines were maintained in the logarithmic phase at 37 °C in a 5% carbon dioxide atmosphere using a RPMI-1640 cell culture medium containing 10% foetal calf serum (Euroclone, Milan, Italy), antibiotics (50 units/mL penicillin and 50 μg/mL streptomycin) and 2 mM l-glutamine.

Human hepatic cancer cell line Huh7 was cultured in MEM supplemented with 10% FCS, L-glutamine, sodium-pyruvate and non-essential amino acids, and with penicillin/streptomycin, at 37 °C in a humidified atmosphere of 5% CO**_2_** and 95% air. For the experiments, the cells were seeded in MEM/10%FCS and then the treatments were continued for an additional 72 h. All *G. diversifolia* extracts were dissolved in DMSO. The final concentration of DMSO did not exceed 0.25% *v*/*v* and the same amount of solvent was added at all experimental points. 

### 4.8. Cell Viability Assay

The growth inhibitory effect towards pancreatic and ovarian tumor cell lines was evaluated by means of MTT assay, as previously described. IC_50_ values were calculated by a four-parameter logistic (4-PL) model.

For the hepatic cells, Sulphorhodamine B (SRB) assay was performed to assess the cell viability after treatment. A total of 8000 cells/well were seeded in a 96-well tray in triplicate [45]. After 24 h of incubation, the cells were treated with different concentrations of compounds. SRB assays were performed after 48 h as previously described [46]. Briefly, the cells were fixed by means of protein precipitation with 50% trichloroacetic acid at 4 °C (50 µL per well, final concentration 10%) for 1 h. After five washing with tap water, the cells were stained for 15 min with 0.4% SRB dissolved in 1% acetic acid (50 µL per well) and subsequently washed four times with 1% acetic acid to remove unbound stain. The plates were air-dried, and bound protein stain was solubilized with 150 µL 10 mmol/L unbuffered Tris base. The optical density was read at 540 nm. Data were expressed as mean ± SD of quadruplicate values of relative absorbance compared to control.

### 4.9. Western Blot Analysis

Cells were washed twice with PBS and lysed with a solution of 50 mM Tris pH 7.5, 150 mM NaCl, 0.5% Nonidet-P40, containing protease and phosphatase inhibitor cocktails (SIGMA, Milan, Italy) for 30 min. on ice. Twenty µg of proteins and a molecular mass marker (Thermo Scientific, Monza, Italy) were separated on 4%–12% SDS-PAGE (BIO-RAD, Segrate (MI) Italy) under denaturing and reducing conditions [47]. Proteins were then transferred to a nitrocellulose membrane using the Trans-Blot**^®^** Turbo™ Transfer System (BIO-RAD). Membranes were washed with Tris-buffered saline-Tween 20 (TBS-T, Milan, Italy), and nonspecific binding sites were blocked in TBS-T containing 5% nonfat dried milk for 60 min at room temperature. Blots were incubated overnight at 4 °C with a diluted solution (5% nonfat dried milk) of the following human primary antibodies: anti LDLR (mouse monoclonal antibody, Millipore clone 2H7.1; dilution 1:1000), and anti-α-tubulin (mouse monoclonal antibody, Sigma clone DM1A; dilution 1:2000). Membranes were washed with TBS-T and then exposed for 90 min at room temperature to a diluted solution (5% nonfat dried milk) of the secondary antibodies (peroxidase-conjugate goat anti-rabbit, and anti-mouse, Jackson ImmunoResearch). Immunoreactive bands were detected by exposing the membranes to Clarity^TM^Western ECL chemiluminescent substrates (Bio-Rad) for 5 min, and images were acquired with an Azure c400 Imaging System (Aurogene**)**. Densitometric readings were evaluated using the ImageLab^TM^ software (Bio-Rad, Image Lab Software 6.0.1).

### 4.10. Statistical Analysis

Statistical analysis was performed using the Prism statistical analysis package, version 5.01 (GraphPad Software, San Diego, CA, USA). p values were determined by Student’s t test. A probability value of *p* < 0.05 was considered statistically significant.

## 5. Conclusions

*Girardinia diversifolia* is a Himalayan nettle employed in traditional medicine for the treatment of several diseases as well as to make fiber. This study was designed to evaluate this plant for its potential pharmaceutical, cosmetic and nutraceutical uses. The methanolic extract presented several activities. Anti-cholinesterase and anti-tyrosinase activities can be mainly related to phytosterols, phytol, and oryzanol, as well as to some of the phenolic constituents. Moderate antioxidant effects are observed, probably due to the low abundance of phenolics and carotenoids, known as highly active antioxidant compounds. The metal chelating activity was significant, showing potential usefulness in the protection of metal based red/ox reactions. An in vitro test of total methanol extract showed a significant cytotoxic effect on BxPC3 and Huh7 cells. *G. diversifolia* extracts were able to reduce the expression of LDLR, and this may explain, in part, their cytotoxic effects on this cell line. Thus, Himalayan nettle can be considered as a valuable source of extracts rich in phytosterols, hydroxycinnamic acids and with potential bioactivities; nevertheless, further studies are needed to assess its efficacy and safety for the development of pharmaceutical, cosmetical or nutraceutical ingredients.

## Figures and Tables

**Table 1:** Identified lipophilic compounds in *G. diversifolia* extract by LC-APCI-MS. Compounds were identified on the basis of literature data (CIT) and injection of authentic standards (*).

RT(min)	[M + H-H_2_O]^+^	Fragments	Identification	mg/g
6.7	425	405-389-365-299-251	Erythrodiol	0.50 ± 0.05
7.8	425	405-389-365-299-251	Uvaol	0.54 ± 0.05
8.5	427	409-391-255-173	Hydroxy cycloartenol	0.25 ± 0.05
9.2	409	391-339-297-269-173	Cycloartenol	0.35 ± 0.05
21.7	395	297-255-241-199-159	Fucosterol *	23.5 ± 0.05
21.8	397	315-299-285-257-243-203-189	γ-Sitosterol *	91.0 ± 0.05
22.5	381	297-255-227-173-159	Brassicasterol *	3.14 ± 0.05
23.1	429	165-137-122-67	α-Tocopherylquinone	n.d.
24.7	383	273-257-243-215-161	Campesterol	32.5 ± 0.05
27.6	397	257-175-161	β-sitosterol *	112.4 ±0.07
29.6	399	316-257-243-190-175-149	Sitostanol *	16.6 ± 0.05
5.07	553		α-Cryptoxanthin	3.9 ± 0.1
5.91	549.5		Carotenoid	3.4 ± 0.1
6.02	545.5	489-435-339	Phytoene	1.3 ± 0.1
6.23	555.1	534-460-442	β-Carotene epoxide	4.0 ± 0.1
6.43	597.5	534-460-442	Zeaxhantin *	6.1 ± 0.1
7.05	553.5	534-460-442	Cryptoxanthin	8.7 ± 0.1
7.58	551.4	535-558-471-444	Ketocarotenoid	3.8 ± 0.1
8.53	551.5	535-558-471-444	Ketocarotenoid	3.2 ± 0.1
9.62	551.5	535-558-471-444	Ketocarotenoid	2.5 ± 0.1
9.78	551.5	535-558-471-444	Ketocarotenoid	1.6 ± 0.1
10.05	537.5	457-445-413	β-Carotene *	1.9 ± 0.1

**Table 2:** Identified polar compounds in *G. diversifolia* extract by LC-DAD-ESI-MS, * indicate comparison with authentic standard.

RT(min)	[M – H]^−^	Fragments	Identification	mg/g
1.8	341	179	Sucrose *	nd
1.9	191	179 85	Quinic acid *	0.138 ± 0.021
2.2	191		Citric acid *	0.235 ± 0.045
2.3	195	177 129 85	Gluconic acid	nd
2.7	353	191 179 135	3-O-Caffeoyl quinic acid	0.056 ± 0.045
3.7	371	209 191 85	*trans*-Syringin (eleuteroside B)*	0.503 ± 0.046
5.2	315	153	Protocatecuic acid glucoside	0.121 ± 0.031
6.3	365	211 153	Protocatecuic ester with sinapoyl alchol	0.103 ± 0.031
6.6	447	357 315 271	Isoorientin	0.090 ± 0.032
7.85	175	157 115 85	Ascorbic acid	2.901 ± 0,031
8.1	371	147 209	Synapoilquinic acid	0.162 ± 0.051
9.2	385	224	Synapoyl acid hexoside isomer 1	0.081 ± 0.028
10.3	447	285 199 243 175	Kaempferol-3-*O*-glucoside *	0.033 ± 0.002
10.9	367	191	5-*O*-Feruloyl quinic acid	0.191 ± 0.029
11.5	385	224	Synapoyl acid hexoside isomer 2	0.151 ± 0.031
11.8	841	779 679 617, 547, 529	3-[Xyl]-28-Glc-11-Hydroxyphytolaccagenin	0.154 ± 0.021
12.0	431	341 311 283	Vitexin*	0.058 ± 0.023
12.8	841	679 517 473 457 437	Scabran G	0.035 ± 0.011
13.6	563	443 413 323	Vitexin-2″*O*-xyloside*	0.171 ± 0.028
14.2	593	473 413 341 311	Vitexin-2″*O*-glucoside*	0.085 ± 0.006
14.3	679	517 473 455 438	Ganoderic acid C2 hexoside isomer 1	0.079 ± 0.007
14.9	679	517 499 473 455 438	Ganoderic acid C2 hexoside isomer 2	0.074 ± 0.007
16.3	679	518 499 473 455 438	Ganoderic acid C2 hexoside isomer 3	0.037 ± 0.007
18.9	517	499 437 304	Ganoderic acid C2	0.038 ± 0.0071
19.4	327	309 291 229	oxo-dihydroxy-octadecanoic acid	0.390 ± 0.008
	[M + H]^+^			
15.4	805	643	Malvidin-3-*O*-glucoside-4-vinyl-catechin	0.032 ± 0.008
8.7	377	243	Vitamin B2	0.122 ± 0.021

**Table 3:** constituents in *G. diversifolia* extract by GC-MS after derivatization.

RT (min)	Identification	MW (Da)	CAS	mg/g
14.2	Phytol	296	102608-53-7	26.72 ± 0.05
18.4	Myristic acid, methyl ester (C16:0)	242	124-10-7	0.70 ± 0.03
23.1	Palmitic acid, methyl ester (C10:0)	270	112-39-0	37.18 ± 0.05
24.3	7-hexadecenoic acid, methyl ester (C16:1)	268	5687-67-3	2.06 ± 0.06
27.6	Stearic acid, methyl ester (C18:0)	298	112-61-8	8.98 ± 0.06
28.7	Elaidic acid, methyl ester (C18:1)	296	112-62-9	8.66 ± 0.06
30.4	Linoleic acid, methyl ester (C18:2 ω-6)	294	112-63-0	21.95 ± 0.08
31.8	Arachidic acid, methyl ester (C20:0)	326	1120-28-1	6.01 ± 0.05
32.3	Linolenic acid, methyl ester (C18:3 ω-3)	292	301-00-8	9.69 ± 0.06
33.5	Heneicosanoic acid, methyl ester (C21:0)	340	6064-90-0	1.22 ± 0.03
35.6	Docosanoic acid, methyl ester (C22:0)	354	929-77-1	2.94 ± 0.03
37.3	Tricosanoic acid, methyl ester (C23:0)	368	2433-97-8	0.65 ± 0.02
38.5	1-docosanol	326	661-19-8	0.36 ± 0.02
39.1	Lignoceric acid, methyl ester (C24:0)	382	2442-49-1	2.42 ± 0.04
42.3	Cerotic acid, methyl ester (C26:0)	410	5802-82-4	0.80 ± 0.02
45.5	Montanic acid, methyl ester (C28:0)	438	55682-92-3	0.52 ± 0.02

**Table 4:** Results of in vitro enzyme inhibition assays on *G. diversifolia* methanolic extract. Values are reported as mean ± SD of three parallel experiments. GALAE: Galantamine equivalent; ACAE: Acarbose equivalent; KAE: Kojic acid equivalent.

Extract	AchE Inhibition (mgGALAE/g)	BuChE Inhibition (mgGALAE/g)	Tyrosinase Inhibition (mgKAE/g)	Amylase Inhibition (mmolACAE/g)	Glucosidase Inhibition (mmolACAE/g)
*G.diversifolia*	4.08 ± 0.21	7.21 ± 0.61	138.14 ± 1.36	1.19 ± 0.05	5.42 ± 0.03

**Table 5:** Results of in vitro antioxidant assays on *G. diversifolia* methanolic extract. Values are reported as mean ± SD of three parallel experiments. GAE: Gallic acid equivalent; RE: Rutin equivalent; TE: Trolox equivalent; EDTAE: EDTA equivalent; na: not active.

Extract	Total Phenolic Content (mgGAE/g)	DPPH (mgTE/g)	ABTS (mgTE/g)	CUPRAC (mgTE/g)	FRAP (mgTE/g)	Metal Chelating (mgEDTAE/g)	Phosphomolybdenum (mmolTE/g)
*G. diversifolia*	24.30 ± 0.15	14.37 ± 0.72	28.33 ± 1.21	105.27 ± 0.65	42.63 ± 0.69	25.70 ± 2.35	2.53 ± 0.25

**Table 6:** Quantitative data on phytosterol in fractionated extracts of *G. diversifolia*, *for phytol, quantitative data were obtained by GC-MS.

Ion	Identification	Hexane Extract (mg/g)	Dichloromethane Extract (mg/g)	Ethyl Acetate Extract (mg/g)
[M + H-H_2_O]^+^425	Erythrodiol	0.35 ± 0.02	2.26 ± 0.04	3.38 ± 0.02
[M + H-H_2_O]^+^425	Uvaol	0.28 ± 0.02	1.46 ± 0.02	1.96 ± 0.04
[M + H-H_2_O]^+^427	Hydroxy cycloartenol	0.24 ± 0.05	5.22 ± 0.03	0.24 ± 0.02
[M + H-H_2_O]^+^409	Cycloartenol	2.61 ± 0.02	5.65 ± 0.07	4.52 ± 0.04
[M + H-H_2_O]^+^395	Fucosterol *	134.39 ± 0.10	165.27 ± 0.11	7.23 ± 0.07
[M + H-H_2_O]^+^397	γ-Sitosterol *	0.92 ± 0.10	230.77 ± 0.07	55.42 ±0.09
[M + H-H_2_O]^+^381	Brassicasterol *	29.14 ± 0.04	32.40 ± 0.09	5.78 ± 0.02
[M + H-H_2_O]^+^429	α-Tocopherylquinone	n.d.		
[M + H-H_2_O]^+^383	Campesterol	31.53 ± 0.07	29.66 ± 0.07	4.36 ±0.06
[M + H-H_2_O]^+^397	β-sitosterol *	417.52 ± 0.15	112.2 ± 0.07	0.20 ± 0.02
[M + H-H_2_O]^+^395	Stigmasterol	70.29 ± 0.21	50.72 ± 0.17	4.31 ± 0.01
[M + H-H_2_O]^+^399	Sitostanol *	6.11 ± 0.05	6.83 ± 0.03	0.06 ± 0.02

**Table 7:** Tests of extracts of *G. diversifolia* on 2008, BxPC3 and Huh7 cell lines (nd: not determined).

	IC_50_ (µg/mL)		
Extract	2008	BxPC3	Huh7
*G. diversifolia*MeOH	47.5 ± 4.1	12.5 ± 3.5	7.2 ± 2.5
*G. diversifolia* Ethyl acetate	nd	nd	19.9 ± 1.5
*G. diversifolia* Cyclohexane	nd	nd	38.8 ± 1.2
*G. diversifolia* Dichloromethane	nd	nd	Not Active

## Supplementary Materials

File with information on plant material is included in supplementary.
