# Supplementary material for: Himalayan Nettle Girardinia diversifolia as a Candidate Ingredient for Pharmaceutical and Nutraceutical Applications—Phytochemical Analysis and In Vitro Bioassays
Source: Molecules. 2020 Mar 29;25(7):1563. doi: 10.3390/molecules25071563 (PMC7180999; doi:10.3390/molecules25071563)
Supplement: Supplementary file 1 [file molecules-25-01563-s001.pdf]

Botanical Information for *Girardinia diversifolia*

Girardinia diversifolia (Link) Friis is a stout, erect, perennial herb. It can grow from 25 to 200 cm height with perennial roots stock. The aerial parts are armed with numerous slender stinging hairs. Its leaves are deeply 3-5 lobed saw-toothed with bristles with broad and palm shaped. Flowers are yellowish, clustered in a panicle, male flowers are white in lower axillary panicles, and female flowers are in upper bristly axillary and terminal panicles. The plant is found on clump and each clump has many stems, whose bark contains fibers strong, smooth and light largely used for preparation of fibers in Nepal. Flowers comes from July to September and fruits from September to November.

In Nepali, the plant is known as “allo" or “chalnesisno” and in English it is commonly known as “Himalayan nettle”. Due to the presence of stinging hairs, it causes irritation on the skin when it is touched (Manandhar, 2002; Baral and Kurmi, 2006; Ghimire et al. 2008).

Girardinia diversifolia is widely distributed in different parts of Nepal. It occurs abundantly in the forests of hills in moist and damp soil at altitude of 1200 to 3000 meters. It is also found in northern India, Bhutan, Sri Lanka, eastward to central China, Myanmar, Malaysia, Indonesia and Africa (Manandhar 2002, ANSAB 2010, Rajbhandari and Rai, 2019).

The plant is largely used in traditional medicine for different diseases like constipation (Thapa, 2012), gastric disorders, chest pain (Rana et al., 2015), rheumatism, tuberculosis (Nath et al., 2011), headache, joints aches (Manandhar,2002; Kunwar et al., 2012), diabetes (Balami, 2004, Gurung et al.,2012, Tamang et al., 2017, Bhattarai and Tamang, 2017), eczema, worm killer and inflamed pancreas (Joshi, 2008; Hasan et al.,2013). Root paste is applied in dog bite. Leaf juice is used to treat in asthma, gastritis, headache, joint pain, tuberculosis (Subedee et al., 2018), snake bites, abdominal pain and indigestion (Bhat et al., 2013), cough, diarrhea (Parajuli, 2013), gonorrhea, delivery problem (Bhatt and Vashishtha, 2008), bone fracture and to conduct smooth delivery (Pandey et al., 2007), internal injury, blood purification (Rokaya et al., 2010), for body warmth and increase hemoglobin in blood (Ghimire et al., 2018). Because of all these uses, *Girardinia diversifolia* has been important source of herbal medicine for rural areas.

It is also famous for the uses of the bast fibre of bark for making varieties of clothing, ropes, mats, sacks and various other domestic implements and in this context it is among one of the most important non timber forest products used for income generation among rural communities in the Himalaya region of

Nepal (Singh and Shrestha, 1985; Singh and Shrestha, 1988; Clarke, 2007; Barakoti and Shrestha, 2009; Pyakurel and Baniya 2011; Gurung et al., 2012; Bhandari, 2019).Young leaves and inflorescences are cooked as a green vegetables. Roasted seeds are pickled (Manandhar 2002; Joshi, 2008, Kunwar et al., 2012). It is also used as leafy vegetables for preparing snacks (Misra et al.,2008)

The present sample was collected from Sindhuligadhi, Sindhuli, central part of Nepal where East India Company was defeated by Nepalese warriors. The collection ranges from 1332 to 1394m and is located between 27^0 16' 43'' N and 85^0 57' 31'' E.

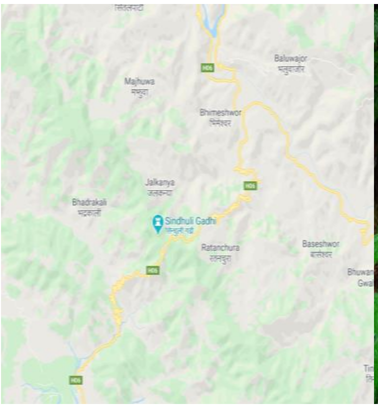

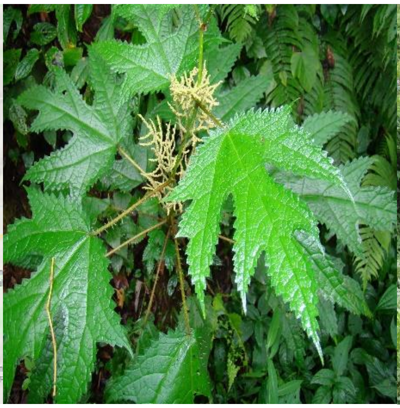
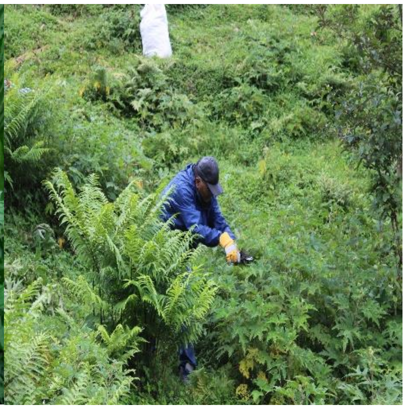

References:

1. ANSAB. 2010. Development and Mobilization of local resource persons (LRPs).Asian Network for sustainable Agriculture and Bioresources, Kathmandu,Nepal.
2. Balami, N.P. 2004. Ethnomedicinal uses of plants among the Newar community of Pharping Village of Kathmandu District,Nepal. Trubhuvan University journal.Vol.24, No.1.
3. Barakoti, T.P. and Shrestha K.P. 2009. Commercial utilization of allo (*Girardinia diversifolia*) by the Rais of Sankhuwasabha for income generation. *Banko Janakari* 18(1):18–24. doi:10.3126/banko.v18i1.2162.
4. Baral, S.R. and Kurmi, P.P. 2006.Compendium of Medicinal plants in Nepal.Rachana Sharma.
5. Bhat,J.A., Kumar,M., Negi,A.K. and Todaria,N.P. 2013.Informants consensus on ethnomedicinal plants in Kedarnath wildlife sanctuary of Indian Himalayas.Department of Forestry and Natural Resources.HNB Garhwal University,Srinagar Garhwal,Uttarakhand,India.

6. Bhattarai, S. and Tamang, R. 2017. Medicinal and aromatic plants: A synopsis of Makawanpur district, central Nepal. *Int J Ind Herbs Drugs* 2(3): 6-15.

7. Bhattarai, S. and Tamang,R. 2017. Medicinal and aromatic plants: A synopsis of Makawanpur district,Central Nepal.Int J Ind Herbs Drugs 2(3):6-15.
8. Bhatti, V. and Vashishtha, D. Indigenous plants in traditional healthcare system in Kedarnath valley of western Himalaya. *Indian J. Tradit. Knowl.* **2008**, *07*, 300–310.
9. Clarke, R.C. 2007. Traditional Nepali hemp textiles. Journal of Industrial Hemp 12(2):97–113. doi: 10.1300/J237v12n02_07.
10. Ghimire, S.K.,Sapkota, I.B.,Oli, B.R. and Parajuli-Rai,R. 2008.Non-Timber Forest Products of Nepal Himalaya: Database of Some Important Species Found in the Mountain Protected Area and Surrounding Regions.WWF Nepal,Kathmandu,Nepal.
11. Ghimire, K.M., Adhikari,M., Uprety, Y. and Chaudhary,R.P. 2018. Ethnomedicinal use of plants by the highland communities of Kailash Sacred Landscape, Far-west Nepal. *Academia Journal of Medicinal Plants* 6(11): 365-378. DOI: 10.15413/ajmp.2018.0171
12. Gurung, A.; Flanigan, H.; Ghimeray, A.K.; Karki, R.; Bista, R.; Gurung, O.P. Traditional knowledge of processing and use of the Himalayan giant nettle (Girardinia diversifolia (Link) Friis) among the Gurungs of Sikles, Nepal. *Ethnobot. Res. Appl.* **2012**, *10*, 167–174, doi:10.17348/era.10.0.167-174.
13. Hasan, M. K., Gatto, P., and Jha, P. K. 2013. Traditional uses of wild medicinal plants and their management practices in Nepal-A study in Makawanpur district. *Int J Med Aromat Plants*, *3*(1), 102-112.
14. Joshi, K.R. 2008. Ethnomedicinal uses of plants: a case study of Sharmoli vdc, darchula district, Nepal. In: *Medicinal Plants in Nepal: An Anthology of Contemporary Research*, P.K. Jha, S.B. Karmacharya, M.K. Chettri, C.B. Thapa and B.B. Shrestha (eds.), pp. 177-186.
15. Kunwar, R.M., L. Mahat, Sharma, L.N., Shrestha, K.P., Kominee, H.and Bussmann, R.W. 2012. Underutilized Plant Species in Far West Nepal. *J. Mt. Sci.* 9: 589–600 DOI: 10.1007/s11629-012-2315-8
16. Manandhar, N. P. 2002. Plants and people of Nepal. Portland: Timber press.
17. Misra,S., Maikhuri,R.K.,Kala,C.P.,Rao,K.S. and Saxena,K.G.2008.Wild leafy vegetables.Astudy of their subsistence dietelic support to the habitants of Nanda Devi Biosphere Reserve, India. Journal of Ethnobiology and Ethnomedicine.4:15,doi:10.1186/1746-4269-4-15.
18. Nath,K.K., Deka,P. and Borthakur, S.K.2011.Traditional remedies of Joint disease of Assam.Indian Journal of Traditional Knowledge,vol.10(3)July,2011.pp.568-571.

19. Pande, P.C.,Tiwari,L. and Pande,H.C.2007.Ethnoveterinary Plants of Uttaranchal-A review.Indian Journal of Traditional Knowledge.Vol.6(3),July2007,pp.444-458.
20. Parajuli, R.R. 2013. Indigenous Knowledge on Medicinal Plants: Maipokhari, Maimajhuwa and Mabu VDCs of Ilam District, Eastern Nepal. *Jour. Dept. Pl. Res. N.* 35: 50-58.
21. Pyakurel, D. and Baniya, A. 2011. NTFPs: Impetus for Conservation and Livelihood Support in Nepal. A Reference Book on Ecology, Conservation, Product Development, and Economic Analysis of Selected NTFPs of Langtang Area in the Sacred Himalayan Landscape. Kathmandu, Nepal: World Wide Fund For Nature (WWF).
22. Rajbhandari, K.R. and S.K. Rai. 2019. A Handbook of the Flowering Plants of Nepal. Volume – 2. Government of Nepal, Ministry of Forests and Environment, Department of Plant Resources, Kathmandu, Nepal.
23. Rana,S.K., Oli,P.S. and Rana,H.K. 2015.Traditional botanical knowledge (TBK) on the use of medicinal plants in Sikles area ,Nepal.Asian Journal of Plant Science and Research,5(11):8-15.
24. Rokaya, M.B.; Münzbergová, Z.; Timsina, B. Ethnobotanical study of medicinal plants from the Humla district of western Nepal. *J. Ethnopharmacol.* **2010**, *130*, 485–504, doi:10.1016/j.jep.2010.05.036.
25. Singh, S.C. and Shrestha, R.R. 1985. A Study of Himalayan Nettle. Kathmandu, Nepal: Research Center for Applied Science and Technology, Tribhuvan University.
26. Singh, S.C. and Shrestha,R.R. 1988. *Girardinia diversifolia* (Urticaceae), a nonconventional fiber resource in Nepal. *Economic Botany* 42(3):445–447.
27. Subedee, B.R.; Chaudhary, R.P.; Uprety, Y.; Dorji, T. Socio-ecological perspectives of Himalayan Giant Nettle (Girardinia diversifolia (Link) Friis) in Nepal. *J. Nat. Fibers* **2020**, *17*, 9–17, doi:10.1080/15440478.2018.1458684.
28. Tamang, R., C. Thakur, D.R. Koirala and N. Chapagain. 2017. Ethno-medicinal Plants Used by Chepang Community in Nepal. *Journal of Plant Resources* 15(1): 21-30.
29. Thapa, S. 2012.Medico-ethnobotany of Magar Community in Salija VDC of Parbat District,Central Nepal.Our Nature 10:176-190.
